# Prevalence of postpartum depression and interventions utilized for its management

**DOI:** 10.1186/s12991-018-0188-0

**Published:** 2018-05-09

**Authors:** Reindolf Anokye, Enoch Acheampong, Amy Budu-Ainooson, Edmund Isaac Obeng, Adjei Gyimah Akwasi

**Affiliations:** 1 Centre for Disability and Rehabilitation Studies, Department of Community Health, Kwame Nkrumah University of Science and Technology, Kumasi, Ghana; 2 School of Public Health, Department of Health Education and Promotion, Kwame Nkrumah University of Science and Technology, Kumasi, Ghana; 3 Methodist University College, Accra, Ghana

**Keywords:** Prevalence, Psychosocial and psychological intervention, Postpartum depression, Ghana

## Abstract

**Introduction:**

Postpartum depression is a mood disorder that affects approximately 10–15% of adult mothers yearly. This study sought to determine the prevalence of postpartum depression and interventions utilized for its management in a Health facility in Ghana.

**Methods:**

A descriptive cross-sectional study design using a quantitative approach was used for the study. The study population included mothers and healthcare workers. Simple random sampling technique was used to select 257 mothers, while a convenience sampling technique was used to select 56 health workers for the study. A Patient Health Questionnaire was used to screen for depression and a structured questionnaire comprising closed-ended questions was used to collect primary data on the interventions for the management of postpartum depression. Data were analyzed using statistical software SPSS version 16.0.

**Results:**

Postpartum depression was prevalent among 7% of all mothers selected. The severity ranged from minimal depression to severe depression. Psychosocial support proved to be the most effective intervention (*p* = 0.001) that has been used by the healthcare workers to reduce depressive symptoms.

**Conclusion:**

Postpartum depression is prevalent among mothers although at a lower rate and psychosocial support has been the most effective intervention in its management. Postpartum depression may affect socialization behaviors in children and the mother, and it may lead to thoughts of failure leading to deeper depression. Frequent screening exercises for postpartum depression should be organized by authorities of the hospitals in conjunction with the Ministry of Health.

## Introduction

Postpartum depression (PPD) is a mood disorder that affects approximately 10–15% of adult mothers yearly with depressive symptoms lasting more than 6 months among 25–50% of those affected [1]. Postpartum depression often occurs within a few months to a year after birth. However, some studies have reported the occurrence of postpartum depression 4 years after birth [2]. Causes of PPD may be physiological, situational, or multifactorial [3].

Major predisposing factors for developing PPD are social in nature usually stressful life events, childcare stress, and prenatal anxiety appears to have predictive value for PPD. In addition, a history of the previous episode of PPD [4], marital conflict, and single parenthood are also predictive [5]. It was believed for a long time that only women from western societies suffered from PPD and that postnatal mood disorders were defined by culture [6]. However, conditions with similar symptoms have also been identified in other countries [7]. Some studies have found the same prevalence of PPD in different societies [8]; however, European and Australian women appear to have lower levels of PPD than women in the United States of America (USA). Women from Asia and South Africa have been identified as being most at risk [9]. The symptoms are similar to symptoms of depression at other times of life, but in addition to low mood, sleep disturbance, change in appetite, diurnal variation in mood, poor concentration, and irritability, women with PPD also experience guilt about their inability to look after their new baby [10]. For most women, symptoms are transient and relatively mild known as postpartum blues; however, 10–15% of women experience a more disabling and persistent form of mood disturbance [11].

More recent evidence suggests that postpartum psychiatric illness is virtually indistinguishable from psychiatric disorders that occur at other times during a woman’s life [12]. Interventions for PPD include pharmacologic interventions, supportive interpersonal and cognitive therapy, psychosocial support through support groups, and complementary therapies. Electroconvulsant therapy has proven effective for mothers with severe PPD [5]. In severe cases of postpartum depression, especially in mothers who are at risk of suicide, inpatient hospitalization may be required [13].

Psychosocial interventions such as support groups have been reported as effective [1, 13]. Beck [1] states that support group attendance can give mothers a sense of hope through the realization that they are not alone. Support groups for couples can teach coping strategies and offer encouragement. They also give couples an opportunity to express needs and fears in a nonjudgmental environment [3].

Interpersonal psychotherapy conceptualizes depression as having three components symptom formation, social functioning, and personal contributions. Emphasis is placed on interpersonal relationships relating to role changes that accompany parenthood rather than on the depression itself. Interpersonal psychotherapy can also be initiated during pregnancy for women who are considered at high risk [13]. Recent research has found that women receiving IPT were significantly more likely to have a reduction in symptoms and recover from PPD than women who did not receive IPT treatment [25].

A study from the United Kingdom found that three brief home-based visits using counseling techniques were effective at accelerating the recovery rate for women suffering from PPD [23].

Prevalence of PPD has been difficult to determine because of the difference in criteria for the time of onset used by the DSM-IV and that used by most epidemiological studies. Prevalence has also been difficult to establish because of underreporting by mothers themselves [2]. It has been estimated that only 20% of women who experience symptoms of PPD report those symptoms to their healthcare providers. Symptoms of PPD are often minimized by both mothers and care providers as normal, natural consequences of childbirth [13]. Evidence has been presented that mothers may also be reluctant to disclose their feelings of depression for fear of stigmatization and fear that their depressive symptoms might be determined as evidence of being a “bad mother”. Cooper et al. [23] reported that “almost half of those independently identified as depressed were not detected as such by their health visitor.

Despite the growing recognition as a global childbirth-related problem, the importance of detecting and treating it has until recently been largely overlooked in practice and it seems that knowledge about this problem is not very high [14]. PPD is a serious social issue due to its consequences, including an increased risk of suicide and infanticide. PPD is often under-diagnosed and untreated; therefore, efforts are needed to improve perinatal mental healthcare [15].

This research was carried out to determine the Prevalence of postpartum depression and interventions utilized by healthcare workers for its management in a Health facility in Ghana.

## Methods

The study was conducted at Komfo Anokye Teaching Hospital in Ghana. The selected hospital is a primary government-owned health facility having several units such as Maternity unit, Reproductive and family planning services, Medical unit, Surgical unit, Adolescent unit, Child Welfare clinic, Outpatient Department, Radiology unit, Accounts, Administration, Medical records, Security, Health insurance unit among others and offer psychiatric services to patients. In this study, a cross-sectional study design with a quantitative approach was used. In cross-sectional studies, investigators do not follow individuals over time. Instead, they look at the prevalence of disease and/or exposure at one moment in time [16]. These studies take a “snapshot” of the proportion of individuals in the population that are, for example, diseased and nondiseased at one point in time. Descriptive cross-sectional studies simply characterize the prevalence of a health outcome in a specified population [16]. This study design was deemed appropriate for this study. The study population included mothers who were within 12 months after delivery because postpartum depression usually affects women within 12 months after giving birth and health workers who were recruited for this study to provide information on the psychosocial and psychological interventions that has been used in the management of postpartum depression at the hospital. The study was conducted within a period of 2 months.

Simple random sampling technique was used to select the mothers. This method selected by chance or none zero mothers for the study and data was collected within a period of 1 month using 5 research assistants. In selecting the respondents for the study, random numbers from a prepared random number table was assigned to names of mothers who were present each day data was collected. The numbers were randomly picked and whichever name that was assigned to the selected numbers that were picked was selected to take part in the study. The Yamane formula for determining samples was used to determine the appropriate sample for the study. A 95% confidence level [The value of (1 − *α*) in standard normal distribution ***z***-table, which is 1.96 for 95%] and a Precision level/sampling error or margin of error of 0.05 or 5% which is the generally acceptable margin of error for social researches [17] were used to calculate for the sample using the equation;

$$n = \frac{N}{{1 + N\left( e \right)^{2} }}$$

*n* represents the sample size to be determined; *N* represents the estimated total population size, and *e* represents the level of precision/sampling error or margin of error. The population of the mothers who had given birth and were within 12 months after delivery at Komfo Anokye Teaching Hospital was estimated to be 451 for the month data was collected.

Therefore;

$$N = 4 5 1$$

$$1 + N \left( e \right)^{2} = { 1 } + { 451 }\left( {.0 5} \right)^{ 2}$$

$$n = \frac{451}{{1 + 451 (.05)^{2} }}$$

$$n = 2 1 2$$

Assuming that 20% will not respond to the questionnaire due to the sensitive nature of the study, 45 (rounded from 42.4) were added to 212. and therefore the total sample size selected amounted to 257 mothers. A convenience sampling technique was also used to select 56 health workers for the study. They were recruited based on their availability and willingness to be part of the study. By the time the investigators completed data collection 56 health workers had availed themselves to be part of the study. The 56 health workers were recruited for this study to provide information on the psychosocial and psychological interventions that have been used in the management of postpartum depression at the hospital.

A Patient Health Questionnaire (PHQ-9) was used to screen for depression at the selected hospital. The PHQ-9 is a 9-question instrument given to patients in a primary care setting to screen for the presence and severity of depression. The PHQ-9 has been validated against in-depth mental health interviews [18, 19] and is reported to be specific (> 86% at scores of > 10) for identification of people with major depressive disorders (MDD) [18, 19].

A structured questionnaire with closed-ended questions was used. The questionnaire was deemed an appropriate instrument for data collection in this study to reap its advantages of cost efficiency, easy administration, and easy quantitative analysis. The questionnaire comprised of four (4) subsections which included questions on the demographic characteristics of respondents; interventions as well as the duration of intervention and influence of interventions on reduction of depressive symptomatology.

**Fig. 1:**
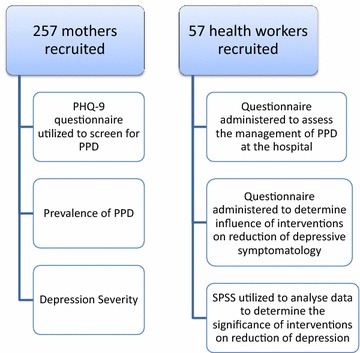
Flow chart

Data were analyzed using both descriptive and inferential statistical tools incorporated in statistical software SPSS version 16.0. To ensure validity and reliability of instruments, the questionnaire was pretested at the Animwaa Hospital, and conflicting issues were resolved before the final data collection (Fig. 1).

## Results

### Demographic characteristics of respondents

The mean age was 27 years while more than half (54%) were married and the majority were Akan’s. Also, more than half (66%) of the respondents had completed JHS/SHS whiles majority (83%) were working in the informal sector as shown in Table 1.

**Table 1:** The demographic characteristics of respondents *Source* Field survey, 2017

Variables	Characteristics	Frequency (*N* = 313)	Percentage (%)	
Age	18–21 years	48	15	
	22–30 years	69	22	
	31–40 years	79	25	
	41–50 years	69	22	Mean = 27.3
	51 years and above	48	15	SD = 8.31
Marital status	Married	115	37	
	Single	74	24	
	Widowed	43	14	
	Divorced	39	12	
	Separated	42	13	
Ethnicity	Akan	155	49	
	Ga/Adagme	56	18	
	Ewe	53	17	
	Gonja	49	16	
Education	No formal education	13	4	
	JHS/SHS	139	44	
	Certificate/diploma	124	40	
	Bachelors	35	11	
	Masters	2	1	
Occupation	Unemployed	22	7	
	Formal	60	19	
	Informal	175	56	
	Midwife	47	15	
	Psychologist	5	2	
	Psychiatrist	4	1	

### Prevalence of postpartum depression

**Fig. 2:**
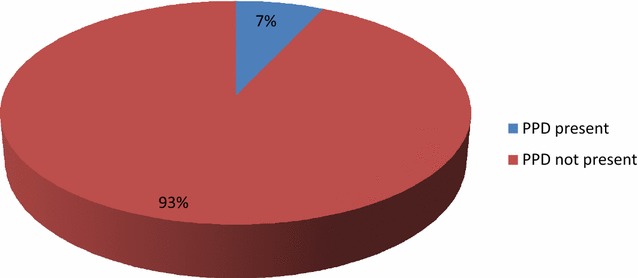
Prevalence of postpartum depression

Figure 2 illustrates the prevalence of postpartum depression among 212 respondents. Out of this total number of respondents, the majority (93%) did not have any indications of postpartum depression (PPD), while 7% had postpartum depression (PPD).

### Depression severity

**Fig. 3:**
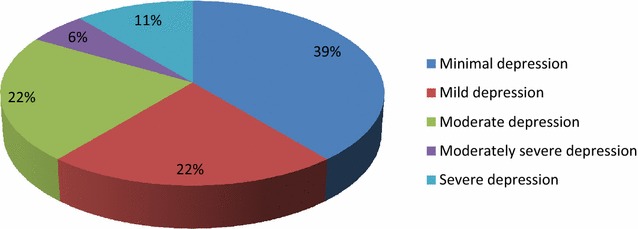
Depression severity

The severity of depression among respondents in the study was further examined and the outcomes are represented in Fig. 3 which shows that 39% out of the total number of respondents had minimal depression; 22% had moderate depression and mild depression, respectively; 6% had moderately severe depression with 11% of the respondents had severe depression.

### Interventions utilized by healthcare workers for the management of postpartum depression

**Fig. 4:**
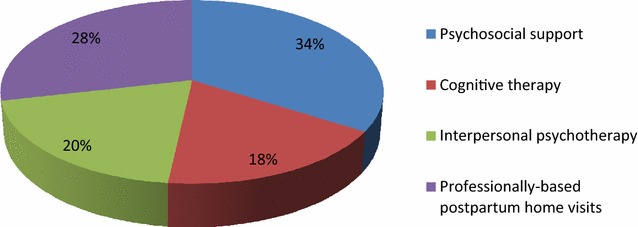
Psychosocial and psychological interventions

Figure 4 indicates the interventions used in the management of postpartum depression among respondents. The most common interventions used in the management of postpartum depression among respondents were psychosocial support (34%), professionally based postpartum home visits (28%), interpersonal psychotherapy (20%), and cognitive therapy (18%).

### Duration of intervention

Table 2 shows the durations of interventions utilized by healthcare workers for the management of postpartum depression. From the table, it is observed that all the interventions were applied up to 6 months.

**Table 2:** Durations of interventions *Source* field survey, 2017

Interventions (*n* = 53)	Less than a month	1–3 months	4–6 months	SD
Psychosocial support	7	10	6	2.08
Cognitive therapy	3	6	3	1.73
Interpersonal psychotherapy	8	3	2	3.21
Professionally based postpartum home visits	1	3	1	1.15

### Influence of interventions on reduction of depressive symptomatology (positive outcome)

From Table 3, cognitive therapy (*p* = 0.14), interpersonal psychotherapy (*p *= 0.356), and professionally based postpartum home visits (*p* = 0.121) had no significant impact on depressive symptomatology reduction, and only psychosocial support (*p* = 0.001) was found to significantly impact on depressive symptomatology reduction.

**Table 3:** Influence of interventions on reduction of depressive symptomatology *Source* field survey, 2017

Interventions (*n* = 53)	Applied	Not applied	Depressive symptomatology reduction	*p* value*
Psychosocial support	23	28	7	0.001
Cognitive therapy	12	39	0	0.14
Interpersonal psychotherapy	13	38	0	0.356
Professionally based postpartum home visits	5	46	1	0.121

### Association between demographic characteristics and depressive symptoms

Table 4 summarizes the result of the univariate and multivariate analysis of the association between demographic characteristics and the presence of depressive symptoms. In both the univariate analysis and the multivariate analysis, ethnicity and occupation had an association with depressive symptoms. Respondents who were Gonja’s were 8.46 times more likely to develop Depressive Symptoms than those in another ethnicity: adjusted Odds Ratio (AOR) = 8.46 [95% confidence interval (CI) 1.57–65.2]. Respondents who were employed were 4.7 times more likely to develop depressive symptoms: adjusted Odds Ratio (AOR) = 4.72 [95% confidence interval (CI) 1.021–14.01].

**Table 4:** Odds ratio with 95% confidence interval for the association between demographic characteristics and depressive symptoms

Variables	Depressive symptoms		Univariate		Multivariate*	
	Present (*n* = 18)	Not present (*n* = 239)	OR (95% CI)	*p* value	AOR (95% CI)	*p* value
Age						
< 40	10	109	1.10 (0.48–2.48)	0.797	0.85 (0.19–3.09)	0.698
> 40	8	130	1.00		1.00	
Marital status						
Married	7	135	1.02 (0.44–2.35)	0.838	1.62 (0.41–5.66)	0.529
Not married	11	104	1.00		1.00	
Ethnicity						
Akan	2	130	1.00		1.00	
Ga/Adagme	1	46	2.01	0.139	1.21 (0.28–6.27)	0.706
Ewe	1	16	1.00			
Gonja	4	47	12.5 (3.54–45.49)	< 0.001	8.46 (1.57–65.2)	0.014
Education						
No formal education	3	10	1.00		1.00	
JHS/SHS	6	109	0.89 (0.41–3.02)	0.797	1.29 (0.35–4.81)	0.122
Certificate/diploma	6	101	2.86 (1.13–20.28)	0.121	1.31 (0.04–2.19)	0.211
Bachelors	3	19	1.00		1.00	
Masters	0	0	1.00		1.00	
Occupation						
Employed	12	223	8.21 (3.12–20.18)	< 0.001	4.72 (1.021–14.01)	0.044
Unemployed	6	16	1.00		1.00	

*OR* odds ratio, *CI* confidence interval, *AOR* Adjusted Odds Ratio

* Mutually adjusted

## Discussion

Findings from this study showed a lower prevalence (7%) of postpartum depression among respondents compared to those found in similar African countries [20–22]. This may be attributed to the instruments used as the PHQ-9 instrument used for this study is different from the other instruments used in the other studies. Respondent’s depressive symptoms varied from being minimal, moderate, mild, moderately severe depression and severe depression. A similar finding was found in South Africa study where prevalence rates of various depressive symptoms were found [23]. The most common interventions used in the management of postpartum depression among respondents were psychosocial support, professionally based postpartum home visits, interpersonal psychotherapy, and cognitive therapy. However, among these interventions the one which had a significant influence on the reduction of depressive symptomatology (positive outcome) was the psychosocial support while the others had minimal influence. Psychosocial interventions are unstructured and nonmanualized and include nondirective counseling and peer support. Psychosocial interventions such as support groups have been reported as effective [1, 13]. The effectiveness of this intervention in the management of postpartum depression (PPD) has been established by Holden [24] in his study 50 women with PPD were randomized to 8 weekly nondirective counseling sessions with a health visitor or routine primary care and it was found that the rate of recovery from PPD for counseling was significantly greater (69%) than that of the control group (38%). From this study, interpersonal psychotherapy intervention and cognitive therapy did not significantly influence the reduction of depressive symptoms. This implies that interpersonal psychotherapy cannot be relied on as an intervention for PPD in the study area. However, the effectiveness of interpersonal psychotherapy in postpartum depression management was confirmed in several studies, including a large randomized trial with a control group [25]. O’Hara et al. randomized 120 women with postpartum depression to receive 12 weekly 60-min individual sessions of manualized interpersonal psychotherapy by a trained therapist versus control condition of a waitlist [25]. The women who received interpersonal psychotherapy had a significant decrease in their depressive symptomatology (measured by Hamilton Depression Rating Scale and Beck Depression Inventory) compared to the waitlist group, as well as significant improvement in social adjustment scores. In another study by Clark et al. [26], 35 women with postpartum depression were assigned to individual interpersonal psychotherapy (12 sessions) versus mother–infant group therapy versus a waitlist condition. Both interpersonal psychotherapy and mother–infant group therapy were associated with greater reduction in depressive symptoms compared to the waitlist conditions. Both studies support the effectiveness of interpersonal psychotherapy as a treatment for PPD, though there is not enough data to suggest a specific benefit to interpersonal psychotherapy compared with other therapeutic modalities. It could, therefore, serve as the first-line treatment, especially for breastfeeding mothers [27].

The study was limited by a smaller sample size, the use of one screening tool for depression among other tools. The study, therefore, missed out on the many other mothers who were not present at the hospital at the time of the study. Moreover, the study failed to determine the prevalence of PPD based on the tools used in other epidemiological studies. However, the Patient Health Questionnaire (PHQ-9) is a multipurpose instrument for screening, diagnosing, monitoring, and measuring the severity of depression. The PHQ-9 incorporates DSM-IV depression diagnostic criteria with other leading major depressive symptoms into a brief self-report tool. While there may be limitations inherent in the study design and methods used, these limitations by no means, compromise the results reported.

## Conclusion

Prevalence of PPD has been difficult to determine because of several factors. The interventions for PPD include pharmacologic interventions, supportive interpersonal and cognitive therapy, psychosocial support through support groups, and complementary therapies. This study found that postpartum depression was prevalent among mothers who were within 12 months after delivery though at a lower rate. Some of the respondents had minimal depression, moderate depression, and mild depression, as well as moderately severe depression, and extremely severe depression. The major predisposing factors for developing PPD are stressful life events, childcare stress, and prenatal anxiety, as well as the history of the previous episode of PPD.

The most-common psychosocial and psychological interventions utilized in the management of postpartum depression were psychosocial support, professionally based postpartum home visits, interpersonal psychotherapy, and cognitive therapy. However, among these interventions, psychosocial support proved to be the most effective intervention as it was reported to have influenced the reduction of depressive symptoms.

Postpartum depression may affect socialization behavior in children and the mother, and it may lead to thoughts of failure leading to deeper depression.

### Recommendations

Frequent screening exercises for postpartum depression should be organized by authorities of the Komfo Anokye Teaching Hospital in conjunction with the Ministry of Health, Ghana Health Service and Nongovernmental Organizations.

The Ministry of Health and Ghana Health Service should collaborate with the National Commission on Civic Education to embark on public education on the effective use of psychosocial support as an intervention for postpartum depression at the various health facilities in Ghana.

## Abbreviations

KATH: Komfo Anokye Teaching Hospital
CBT: cognitive therapy
MDD: major depressive disorders
PHQ-9: Patient Health Questionnaire
ECN: early childhood nurses
PPD: postpartum depression
